# IL17 Mediates Pelvic Pain in Experimental Autoimmune Prostatitis (EAP)

**DOI:** 10.1371/journal.pone.0125623

**Published:** 2015-05-01

**Authors:** Stephen F. Murphy, Anthony J. Schaeffer, Joseph Done, Larry Wong, Ashlee Bell-Cohn, Kenny Roman, John Cashy, Michelle Ohlhausen, Praveen Thumbikat

**Affiliations:** Department of Urology, Feinberg School of Medicine, Northwestern University, Chicago, Illinois, United States of America; Cedars-Sinai Medical Center, UNITED STATES

## Abstract

Chronic pelvic pain syndrome (CPPS) is the most common form of prostatitis, accounting for 90–95% of all diagnoses. It is a complex multi-symptom syndrome with unknown etiology and limited effective treatments. Previous investigations highlight roles for inflammatory mediators in disease progression by correlating levels of cytokines and chemokines with patient reported symptom scores. It is hypothesized that alteration of adaptive immune mechanisms results in autoimmunity and subsequent development of pain. Mouse models of CPPS have been developed to delineate these immune mechanisms driving pain in humans. Using the experimental autoimmune prostatitis (EAP) in C57BL/6 mice model of CPPS we examined the role of CD4+T-cell subsets in the development and maintenance of prostate pain, by tactile allodynia behavioral testing and flow cytometry. In tandem with increased CD4+IL17A+ T-cells upon EAP induction, prophylactic treatment with an anti-IL17 antibody one-day prior to EAP induction prevented the onset of pelvic pain. Therapeutic blockade of IL17 did not reverse pain symptoms indicating that IL17 is essential for development but not maintenance of chronic pain in EAP. Furthermore we identified a cytokine, IL7, to be associated with increased symptom severity in CPPS patients and is increased in patient prostatic secretions and the prostates of EAP mice. IL7 is fundamental to development of IL17 producing cells and plays a role in maturation of auto-reactive T-cells, it is also associated with autoimmune disorders including multiple sclerosis and type-1 diabetes. More recently a growing body of research has pointed to IL17’s role in development of neuropathic and chronic pain. This report presents novel data on the role of CD4+IL17+ T-cells in development and maintenance of pain in EAP and CPPS.

## Introduction

Symptomatic presentation of prostatitis accounts for 8% of all urologist visits and an estimated 1% of all primary care visits in the US and is categorized into five subsets. Of these Chronic pelvic pain syndrome (CPPS) is the most common, accounting for 90–95% of all prostatitis diagnoses [1,2]. The etiology of CPPS is unknown but it is thought to involve pro-inflammatory and autoimmune mechanisms [3]. The syndrome is characterized by the lack of bacterial association with pain symptoms and is subdivided into IIIa and IIIb based on the presence of inflammatory infiltrate (>10 cells/high powered field) in the expressed prostatic secretion (EPS) of patients [1,2]. Given its estimated prevalence of up to 15% of the male population, there is a growing need for a more defined understanding of the mechanisms underlying symptoms [1,4]. Experimental autoimmune prostatitis (EAP) is a non-infectious autoimmune driven murine model of CPPS and is induced by subcutaneous injection of rat prostate antigen and adjuvant into mice followed by weekly behavioral testing for development of pain. Using this model our laboratory has previously shown that mast cells are crucial mediators of development of EAP induced pain in NOD mice [5] and that mast cell tryptase can mediate pain via the PAR2 receptor in C57BL/6 mice [6]. While these studies have been informative in terms of understanding more deeply the crosstalk between activated mast cells and the nervous system, little is known about the role of the adaptive immune response in EAP induced pain. Studies using the uropathogenic *E*.*coli* (CP-1) infection murine model of CPPS have however, demonstrated an important role for Th17 T-cells in mediating prostatic autoimmunity and pelvic pain [3]. This model differs significantly from the EAP model as induction of pain upon CP-1 infection only occurs in the genetically susceptible NOD background [7,8] and not in C57BL/6 mice. IL17 producing cells have been implicated in a wide variety of autoimmune disorders including inflammatory bowel disease [9,10], type-1 diabetes [11], multiple sclerosis [12–14] and rheumatoid arthritis [15]. Multiple models of neuropathic pain have now also shown a role for IL17 in mediating pain and providing new avenues for development of therapeutics in chronic pain syndromes. [16–18].

The objective of this study was to examine the role of CD4 T-cell subsets, specifically Th17 cells, in development of pain in EAP and investigate whether these observations correlate with the immunological profile of human CPPS. We investigated whether prostatic expression of IL17 is increased in EAP mice and if this cytokine is involved in either early initiation of pain or solely in maintenance of chronic pain. Furthermore we also examined levels of cytokines and chemokines in the EPS of CPPS patients and whether there were any relationships between expression level and symptom severity. Using the EAP model we then attempted to corroborate these findings in an animal model. It was hoped that the data resulting from these experiments would give novel immunological insight into the pathogenesis of CPPS while also highlighting the necessity for increased research into the role of the adaptive immune response in mediating prostate derived pelvic pain.

## Materials and Methods

### Animal use

All animal experiments were performed in accordance with standard laboratory protocols under the remit and approval of the IACUC office and the monitoring systems at Northwestern University. C57BL/6 were given EAP as previously reported [19], briefly, subcutaneous injection of rat prostate at a 1:1 ratio with TiterMax adjuvant, control mice were untreated. Pain responses were assessed by tactile allodynia using Von Frey force filament behavioral testing, every seven days post treatment unless otherwise stated. Results were displayed as percent response change above baseline (baseline is testing result prior to treatment) and as an individual score per group per individual filament. After 28 days mice were sacrificed, by an overdose of Isoflurane and euthanasized with cervical dislocation under anesthesia to minimize pain and suffering, tissues removed and dissociated using 1640-RPMI (Corning) containing 10% FCS (Hyclone), 0.1ul/ml DNase (Thermo-scientific) and 1ug/ml collagenase D (Roche) for 2 hours at 37C with shaking. Single cell suspensions were then made using 0.2um mesh membranes, for iliac tissue single cell suspensions were made without necessity for collagenase treatment. Blockade of IL17 was performed, therapeutically; by intraperitoneal injection with 100ug anti-IL17 (R&D Systems MAB421) at Day 10 post-EAP treatment and pain assessed everyday for three days and followed until Day 20. Prophylactic treatment with anti-IL17 antibody (R&D Systems MAB421) was performed by intraperitoneal injection of antibody or normal rat IgG control (R&D Systems 6001A) one day prior to EAP induction and weekly one day prior to behavioral testing. Tissues for QRTPCR analysis were homogenized following removal and Trizol (Invitrogen) RNA extraction performed as above followed by cDNA synthesis.

### Patient Sample Collection

Appropriate National Institute for Health- Chronic Prostatitis Symptom Index (NIH-CPSI) questionnaire data EPS and voided bladder 3 (VB3) samples were collected by Dr A. Schaeffer and colleagues at the Dept. of Urology Outpatient Clinic at Northwestern Memorial Hospital, Chicago IL (NCT01676857). The written consent procedure was reviewed by the Northwestern University Institutional review board Panel D and approved with the IRB number STU00030121. The Northwestern University Institutional review board Panel D specifically approved this study with IRB number STU00030121. All participants provided written informed consent to participate in this study. The questionnaire has been designed specifically to assess patient scored symptoms of CPPS and divides score into three areas, Pain, Urinary and Quality of Life. VB3 samples were centrifuged at 300g for 4 min at 4C and supernatant removed and stored at -80C. The pellet was re-suspended and incubated for 4.5hrs in T-cell activation buffer (1640-RPMI (Corning), 10% FCS (Hyclone), 1% Pen/Strep (Cellgro), 100ng/ml PMA (Sigma), 750ng/ml Ionmycin (Sigma) and 0.66nl/ml Golgi Stop (BD), following incubation cells were fixed overnight in 4% paraformaldehyde and stained for intracellular T-cell markers, IFNγ, FoxP3 and IL17. Samples were analyzed by flow cytometry on a benchtop Accuri C6 cytometer. EPS samples were immediately stored at -80C for future analyses.

### Cytokine Array

A multiplex cytokine array was performed using EPS samples from 25 IIIb patients and 8 controls; IIIb patients defined by absence of infiltrate in EPS and control subjects as healthy volunteers with no history of pelvic pain. Levels of 41 chemokines and cytokines were assayed using the Milliplex MAP kit (Millipore) followed by luminex analysis according to manufacturers instructions. Protein content of EPS was normalized against total protein content, as determined by Bicinchonic Acid Assay (BCA) (Pierce).

### QRTPCR

RNA was isolated from appropriate tissues using Trizol (Invitrogen) and cDNA was synthesized using qScript Supermix (Quanta) according to manufacturers instructions. QRTPCR was performed using perfeCTa qPCR Supermix (Quanta) and run on the CFX Connect (Biorad) platform. Primers were designed using the online NIH curated primer blast tool. A full table of primer sequences is included in supplementary materials. ddCT calculations were performed in Excel and analyzed using GraphPad Prism software.

### Immunoblotting

Protein lysates from murine prostates were generated using NP-40 lysis buffer (Life Technologies) and normalized using BCA assay (Pierce). SDS-PAGE was performed using the BioRad system and Criterion (BioRad) pre-cast 4–12% gels. Immunoblotting was performed using rabbit anti-mouse IL7 antibody (AbCam ab119498) and B-tubulin (Cell Signalling Technology #2128) developed using Supersignal West chemiluminesence kit (Pierce). Densitometry was performed using ImageJ, statistical analyses was performed using GraphPad Prism software.

### Immunohistochemistry

Formalin fixed-paraffin embedded mouse prostate sections were rehydrated in xylene (VWR), followed by decreasing dilutions in ethanol (100%, 75%, 50% and 25%). Following which samples underwent antigen retrieved by boiling for 20mins in 10mM Sodium Citrate Buffer, pH 6.0. Sections were then transferred to PBS (Gibco), excess liquid removed and staining performed using rabbit anti-mouse IL7 antibody (AbCam ab119498) followed by secondary staining with the ImmunoCruz ABC rabbit staining kit (Santa Cruz) as per manufacturers instructions. Sections were photographed using the Leica CTR/MIC DMLA light microscope.

### Flow cytometry analyses

Flow cytometric analyses of T-cell markers was performed using the following antibodies, mouse samples (PerCp-CD4 (Biolegend), FITC-CD25, PE-FoxP3, FITC-IFNg, APC-IL17 (eBiosciences), or human samples (PE-IL17, Alexa647-FoxP3, (Biolegend), PerCpCy5.5-CD4, FITC-IFN-g (eBiosciences) on an Accuri benchtop C6 cytometer. For all analyses unless otherwise stated, samples were gated on lymphocyte populations based on size, as assessed by SSC and FSC, followed by gating for CD4 positivity. Intracellular staining was performed by fixation and permeabilization using eBioscience Fix-Perm Intracellular staining buffers (Cat. Num 8222–49 and 8333–56). Staining was performed for 1 hour at room temperature followed by washing in FACS buffer (2% FCS (Hyclone), in PBS (Gibco)) Analyses were performed using FlowJo and data statistically tested using GraphPad Prism software, with tests described in respective figure legends.

### Statistical Analyses

Statistical analyses were performed using GraphPad Prism software. Multiplex cytokine array analysis was performed using a combination of linear regression and correlation (Pearson) between each cytokine and patient-reported pain, urinary, QOL and total scores. Total differences were determined between patients and controls using unpaired T-test, with those showing significance subjected to ROC (Receiver Operator Characteristic) analyses. ROC analysis for combined efficacy of cytokines as diagnostic tools was performed by bilinear logistic regression in SAS followed by ROC analyses using outcome values. Expression differences at cDNA and protein levels were assessed by unpaired T-test, with experimental values normalized against appropriate controls, with n = 5 mice per group in experimental duplicate and biological triplicate (cDNA synthesis). Flow cytometric samples were compared using one-way ANOVA with multiple comparisons against control untreated samples. All datasets were treated as normal, unless otherwise stated, assessed by median to mean agreement.

## Results

### Increased IL17 expressing CD4 cells in EAP mice

**Fig 1:**
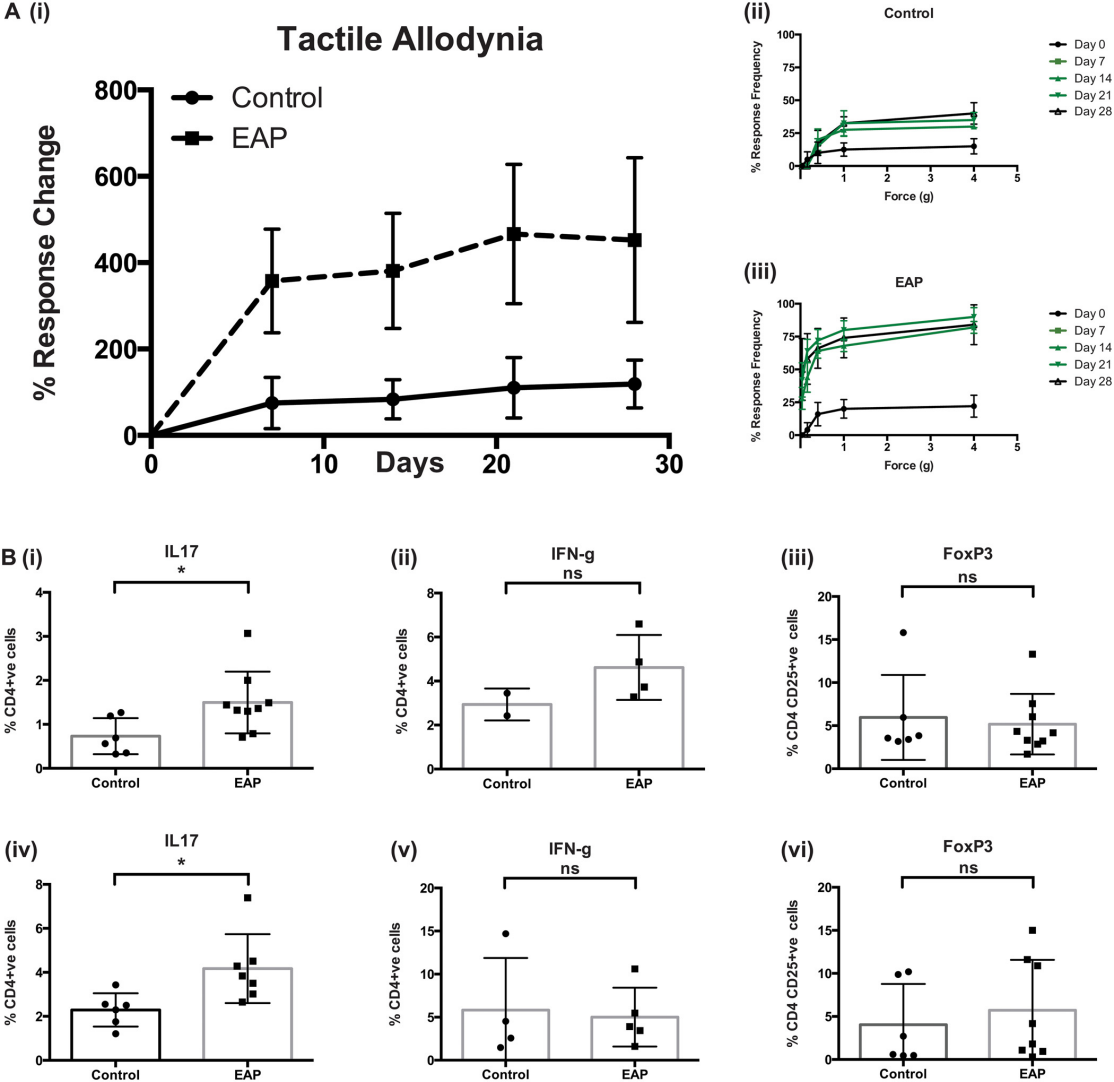
EAP induces pain and expression of IL17 in C57BL/6 mice. A(i). EAP induces pain as assessed by tactile allodynia Von Frey testing in C57BL/6 mice. Pain is depicted as increased percentage response frequency above baseline every 7 days for 28 days. A(ii-iii), Responses frequencies to filaments on increased force showing increased response as early as day 7 post-EAP induction. B. Flow cytometry staining for T-cell extra and intracellular markers showing increases in CD4+ve IL17+ve cells in both EAP prostate, (i) and iliac lymph node, (iv) tissues compared to controls. Staining for CD4+ve IFNγ (ii, v) cells and CD4+ve CD25+ve FoxP3+ve (iii, vi) show no significant changes between EAP group and controls. Mean +/- SEM is shown, gating was performed as depicted in S1 Fig Flow cytometry experiments are the average of at least two experiments with N = 4/5 mice per group. * = p<0.05, ** = p<0.01.

Mouse models of CPPS have been developed to understand the mechanisms driving pain and inflammation in humans. EAP is one such model where prostatitis and subsequent pelvic tactile allodynia is induced upon subcutaneous injection of rat prostate antigen behind the right shoulder of C57BL/6 mice. Tactile allodynia (metric for pain) usually peaks 21 days post injection, measured using Von Frey filament behavioral testing, as described previously [5,19]. Fig 1A(i), depicts a graphical output of the Von Frey testing, with tactile allodynia responses increasing every 7-days out to day 28, and responses to individual filaments, Fig 1A(ii-iii), of increasing force also showing significant differences between control and EAP animals. The local adaptive immune response of the prostate and draining iliac lymph nodes to EAP induction has not been defined. To assess this we performed flow cytometry on single cell suspensions of prostate and iliac lymph node tissues and compared expression of Th1, Th17 and T-reg cell markers between control and EAP animals. Fig 1B(i-vi) demonstrates a statistically significant increase in CD4+ve IL17+ve cells in both tissues examined. Intracellular staining for IFNγ revealed a similar trend that did not reach significance in either tissue, flow cytometry gating is depicted in S1 Fig Levels of CD4+ve CD25+ve FoxP3+ve cells appeared unchanged upon EAP induction. These data indicate that CD4+ IL17 producing cells are increased as part of the local prostatic response to subcutaneous antigen in C57BL/6 mice. Similar analyses using splenic tissues from these mice demonstrate that these immunological changes were not detectable systemically, S2 Fig.

### IL17 is essential for development of pain in EAP

**Fig 2:**
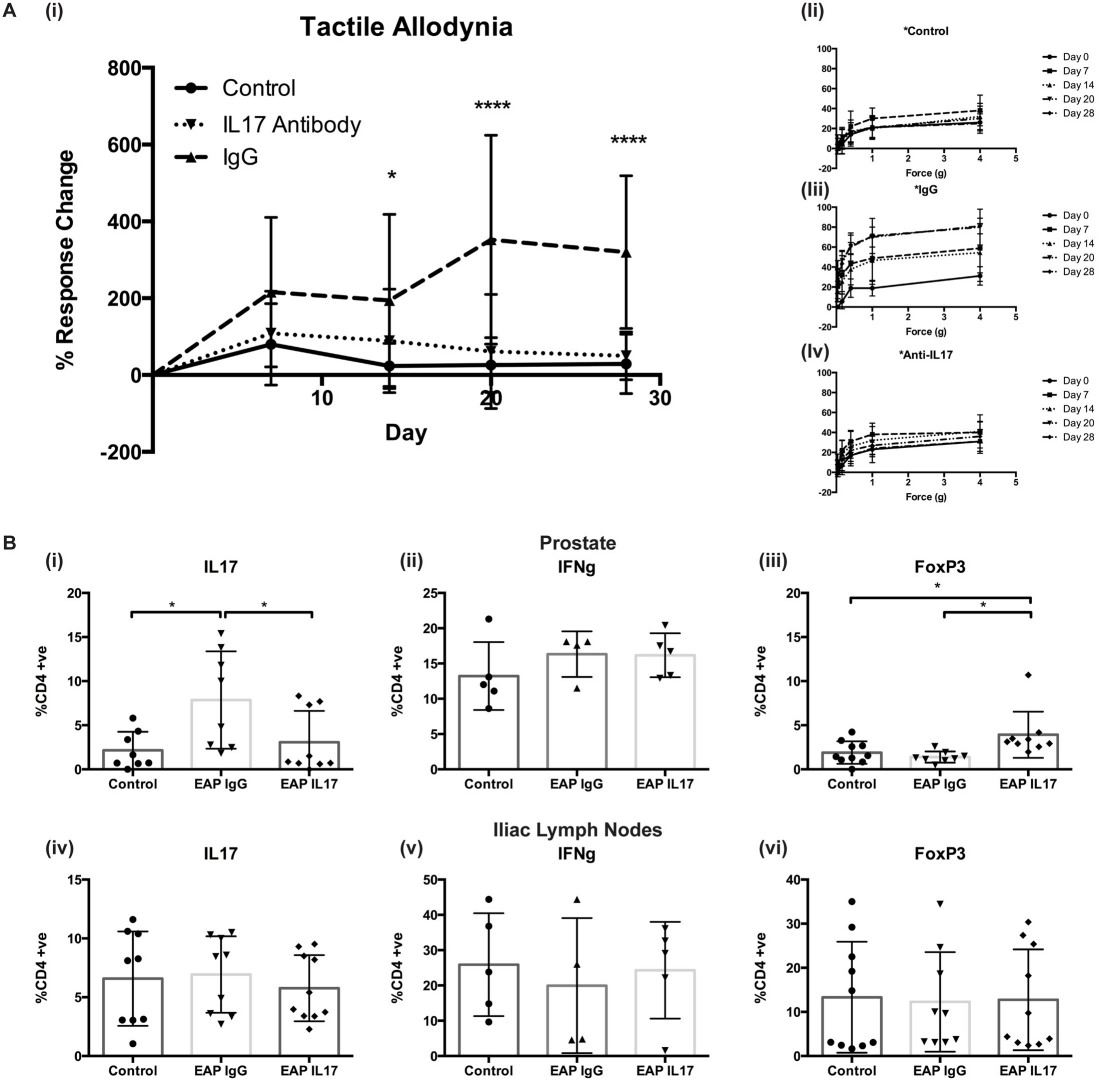
IL17 is essential for development of EAP induced pain in C57BL/6 mice. A. anti-IL17 blocking antibody or an IgG isotype control were given by i.p. injection one day prior to induction of EAP and one day prior to tactile allodynia testing every 7 days for 28 days. (i) Percentage response frequency curves demonstrating no increase in pain above baseline in anti-IL17 treated group compared to IgG control. (ii-iv) Response frequency to individual filaments for each mouse group. B. Flow cytometry analyses for T-cell markers in prostate and iliac lymph node tissues from these mice for (i, iv) (ii, v) CD4+ve IL17 +ve, (iii, vi) CD4+ve IFNγ and CD4+ve CD25+ve FoxP3+ve cells. Mean +/- SEM, gating strategy is outlined in S1 Fig Statistics were performed in Prism software using, Two-way ANOVA for response change analyses, and unpaired student’s T-tests for flow, averages of two separate experiments N = 4/5 mice per group displayed for both. * = p<0.05, **** = p<0.0001.

Given the data suggesting a role for Th1 and Th17 cells in mediating pain and inflammation in EAP and our previously published data demonstrating increased pain upon induction of pain by CP-1 infection in IFN-γKO mice [3], we next aimed to determine whether IL17 was important for either initiation or maintenance of prostatitis. In order to address this we treated mice with an anti-IL17 antibody or an IgG control 10 days following EAP induction and tested mice everyday for 3 days, S3 Fig These data demonstrated no decrease in pain upon treatment with the antibody compared to the IgG control, as far out as day 20. Thus, indicating that blockade of IL17 activity following EAP induction was not sufficient to ameliorate pain. To assess the necessity of IL17 in development of EAP we treated mice prophylactically with an IgG control or an anti-IL17 antibody one day prior to EAP induction. Fig 2A(i), demonstrates an inability of EAP to induce pain, as assessed by tactile allodynia, following disruption of IL17 activity. Individual responses to increasing force of Von Frey filaments, Fig 2(ii-iv) also demonstrated a significant decrease, matching control animals, to pain in the absence of IL17. Taken together these data suggest that IL17 is fundamental to development of EAP and subsequent pain in C57BL/6 mice but that it is not sufficient for maintenance of pain following previously established auto-immunity.

In order to determine the immunological consequence of prophylactic treatment with anti-IL17 we performed flow cytometry on single cell suspensions from prostate and iliac lymph node tissue from EAP treated and control mice. Fig 2B(i-vi), demonstrates a similar level of CD4+ve IL17+ve T-cells in prostates of untreated controls and mice treated with the antibody and EAP. Mice treated with the IgG control antibody show increased levels of IL17+ve CD4 cells mirroring data from previous investigations, Fig 1B(i-vi). Treatment with the IgG antibody appeared to alter differences in CD4+ve IL17+ve cells at the iliac lymph node without decreasing tactile allodynia responses in these mice. Interestingly ablation of IL17 function resulted in an increase in FoxP3+ve cells in the prostates of these mice, suggesting that the IL17-T-reg T-cell subset axis is important for development of autoimmunity and pain in EAP and that enhancing CD25+FoxP3+ CD4 T-cells may serve as a potential therapeutic for pain amelioration. Again these responses were shown to be local as no significant effects were observed in splenic tissues of these mice, S4 Fig.

### IL7 is correlated with increased patient pain and symptom scores

**Fig 3:**
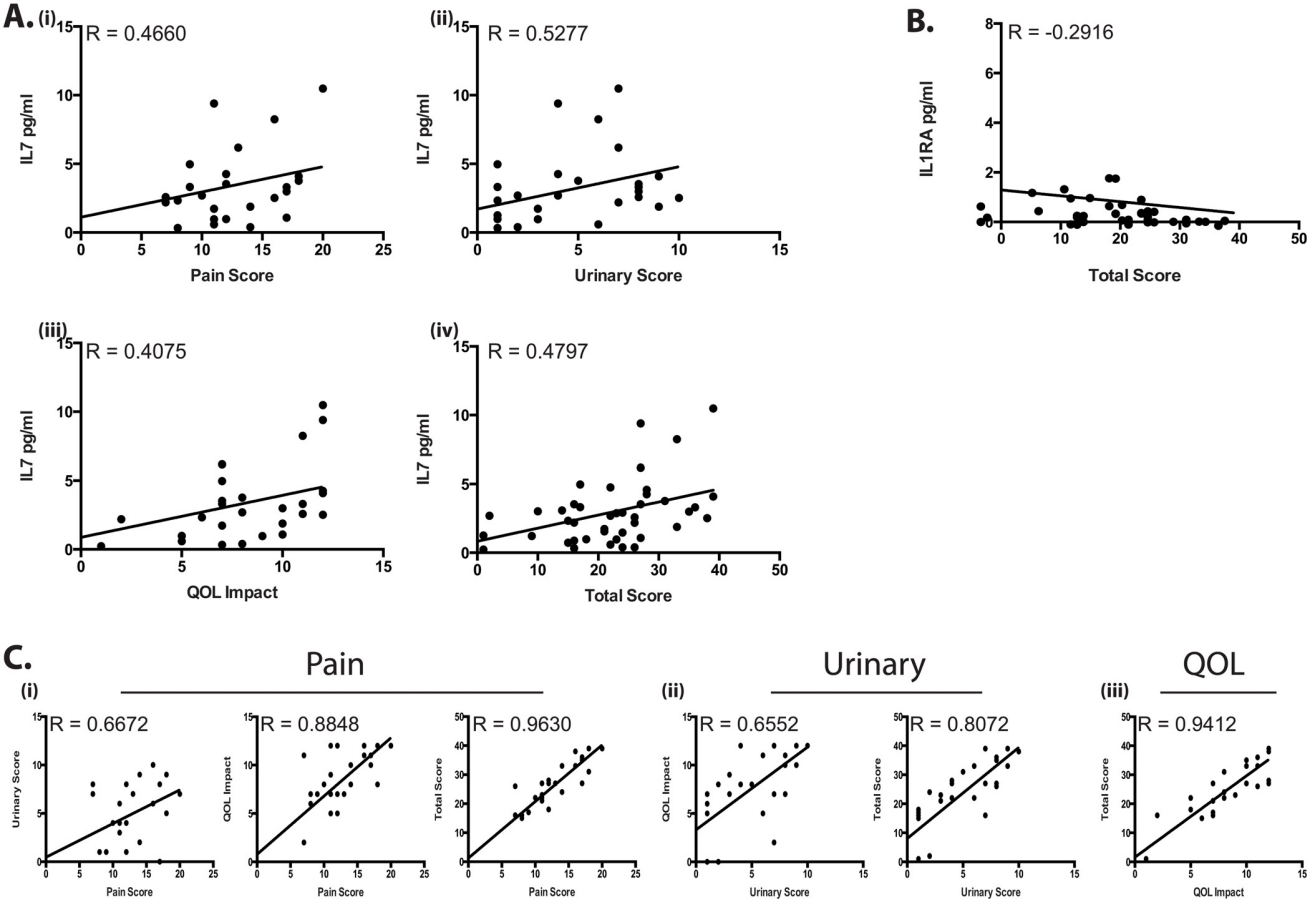
Increased IL7 in EPS is correlated with patient symptom scores. A (i-iv) Positive correlation between increasing patient scores and levels of IL7 in EPS. B Negative correlation between Total score and IL1RA. C (i-iii) Positive correlations between each patient score metric. * = p<0.05, ** = p<0.01.

To determine the immunological profile of chronic pelvic pain syndrome patients, EPS samples from patient and controls (patient demographics in S1 Table) were run on a multiplex cytokine bead array and measured for levels of 41 immunological analytes. This investigation focused solely on IIIb patients in order to determine the immunologic changes leading to pain and symptom development rather than those potentially related to increased infiltration of leukocytes, such as the previously reported, MCP1 and MIP1α [20,21]. These expression data were then correlated in Prism with patient and control information from NIH-CPSI questionnaires. Statistical analysis of the array revealed only one cytokine to be consistently correlated with patient report edmetrics, IL7. Fig 3A(i-iv). These linear regression and correlation (Pearson) plots demonstrate that as patient score, pain, urinary symptoms, quality of life (QOL) impact and total score increased, physiological levels of IL7 measured in EPS also increased. IL7 is a well-defined cytokine involved in early T and B-cell maturation and as such is an essential driver of T-cell mediated immunity [22–24]. IL7 has a known role in the development and maintenance of IL17 producing T-cells and polymorphisms in its cognate receptor IL7R (CD127) have been shown to be associated with a variety of autoimmune disorders including, multiple sclerosis (MS) [12,23,25,26] and type-1-diabetes (T1D) [27,28]. More recently IL7 has been shown to mediate T-cell subset differentiation by driving CD4+veCD25+ve naïve and activated memory T-cells to a pro-inflammatory Th17, IL17 producing T-cell phenotype. These switches have been associated with treatment with exogenous IL7 (HIV patients) and increases in ATP receptor activation, specifically the P2X family [24]. Furthermore it has been shown to alter the suppressive effects of T-regulatory cells and to drive expansion of autoreactive T-cells [29]. These analyses also revealed a statistically significant negative correlation between levels of IL1RA and Total patient scores (Fig 3B). In contrast to IL7’s potential role in mediating pro-inflammatory T-cell immunity, IL1RA is an IL1 receptor antagonist and has been shown to inhibit both IL1α and IL1β, and dampen IL1 mediated pro-inflammatory signaling [30–32]. Taken together, these correlations highlight the role of the adaptive immune system in mediating the severity of pain, urinary and quality of life impact in CPPS patients. In tandem with this our data indicates that even in the absence of infiltrating leukocytes the adaptive T-cell response is mediating increases in prostate pain. Fig 3C(i-iii) demonstrates the relationships between each of the patient metrics assessed, outlining strong positive correlations between each of the measured symptoms, indicating perhaps an interlinked mechanism underlying both pain score and urinary symptoms.

### Increased IL7 and GROα in CPPS patients

**Fig 4:**
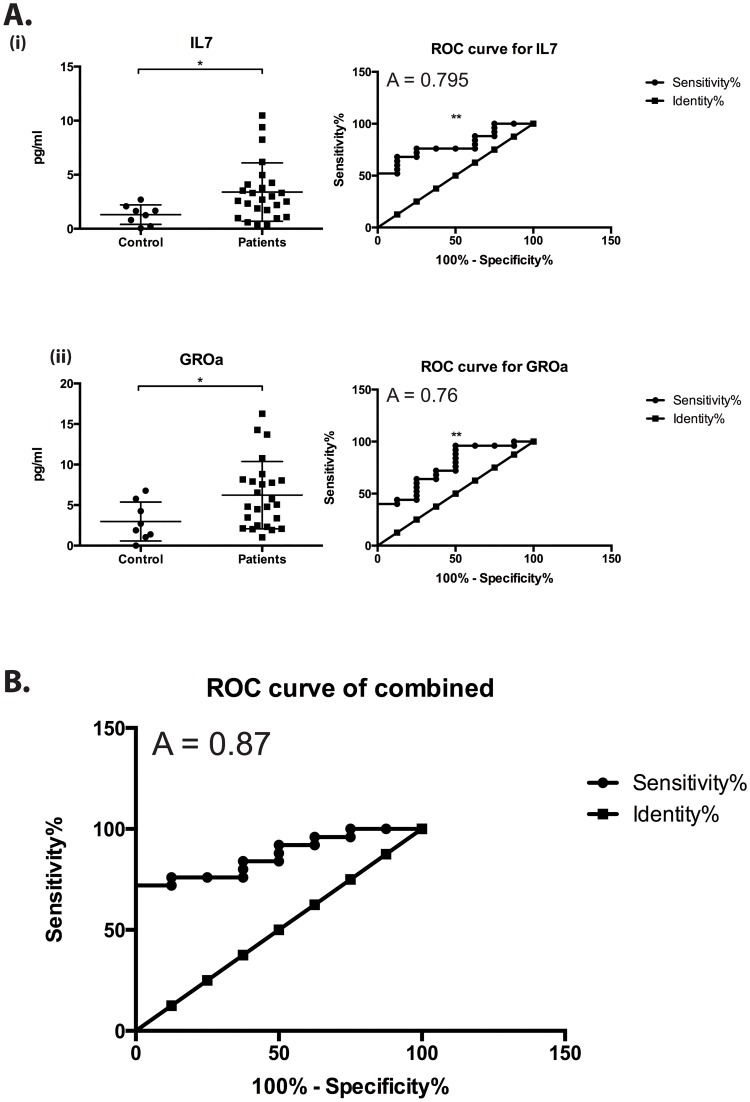
CPPS patients have higher levels of IL7 and GROα. A (i-ii) Total differences between control and CPPS patient EPS samples with Receiver Operator Curves (ROC), mean +/- SEM are shown. B. Combined ROC curve demonstrating increased diagnostic power of both analytes together. Statistics performed in SAS and Prism using, linear regression, bilinear logistic regression, Pearson Correlation, unpaired T-test, and ROC analyses, A = area under curve, * = p<0.05, ** = p<0.01.

Total differences between patients and controls were also examined for each analyte and these revealed significant increases in levels of IL7 and GROα, a neutrophilic chemo-attractant [33]. Fig 4A(i-ii). No significant decreases were observed for any protective or anti-inflammatory analyte. ROC analyses were then performed to test the efficacy of each significantly increased analyte to act as a diagnostic tool. This revealed area under the curve values of 0.795 and 0.76 respectively, with sensitivity at 96% and specificity at 25% for values above 0.36pg/ml and 1.211pg/ml respectively. Furthermore, and more usefully, combining these two markers, using logistic regression only increased diagnostic power, with an area under the curve value of 0.87, Fig 4B. These data indicate that T-cell signaling, development and maturation is fundamentally linked to increasing levels of pain associated patient morbidity in CPPS IIIb and identify IL7 and GROα as potential diagnostic markers of CPPS and specifically IL7 as a metric of pain severity. Although the received operator curves for both increased analytes indicate the potential for their use as biomarkers the small patient populations and high specificity values must be taken into account before robust conclusions are drawn from these data.

### IL7 expression in EAP

**Fig 5:**
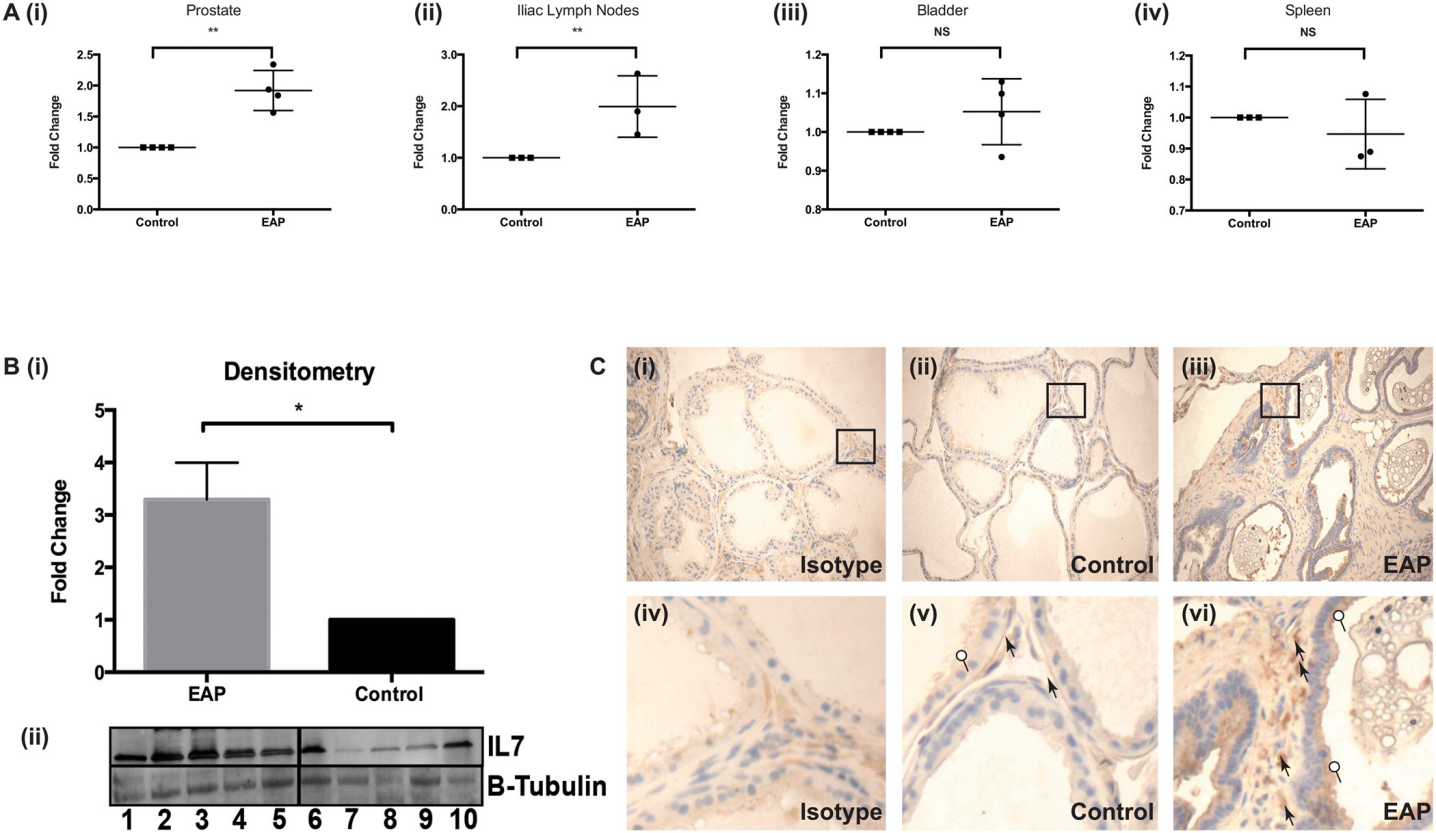
IL7 expression is increased in a prostate specific manner in EAP mice. A (i-iv) QRTPCR of IL7 from murine splenic, bladder, prostate and iliac lymph tissues. B. Immuno-blotting and densitometry for IL7 using prostate tissue lysates. C. Immuno-histochemical staining for IL7, (i-ii) Isotype staining on EAP prostate (iii-iv) IL7 staining on control prostate and (v-vi) IL7 staining on EAP prostate, circle; epithelial, arrowhead; stromal cell staining. Immunoblot densitometry performed in ImageJ using area under the curve values followed by a T-test. Mean +/- SEM are shown, statistics performed in Prism using unpaired T-test, QRTPCR samples normalized to relevant controls with L19 as housekeeping gene and displayed as fold change, performed in technical duplicate and biological triplicate with N = 4/5 mice per group. * = p<0.05, ** = p<0.01.

Although we did not see significant increases in IL17 in our patient population we sought to examine whether IL7, a homeostatic mediator of these cells, was increased in the EAP model. Thus we examined levels of expression of IL7 in mice upon induction of EAP. QRTPCR was performed for IL7 using cDNA synthesized from prostatic, bladder, iliac lymph node and splenic tissues and levels compared between control and EAP animals. Fig 5A(i-iv) shows significant differences between IL7 mRNA level in both prostates and iliac lymph nodes of EAP mice compared to controls with no such differences observed in either bladder or splenic tissue. These results indicate that the observed increases in IL7 expression are prostate specific, given that the iliac lymph nodes are the main draining lymph node of the mouse prostate. Furthermore, these differences were confirmed at the protein level by immunoblot using prostate tissue lysates, representative immune-blot Fig 5B(i), and quantified by densitometry, Fig 5B(ii). In order to assess which tissue within the prostate was the source of IL7, we performed immunohistochemical analyses. This revealed increased expression of IL7 in both stromal and epithelial tissues of the dorsolateral prostate, Fig 5C(i-vi), between control and EAP animals. These data not only strongly implicate IL7 in mediating murine EAP but also more importantly demonstrate the relevance and efficacy of using murine models to delineate immunological mechanisms underlying human CPPS.

### Increased IL7 expression is dependent on IL17

**Fig 6:**
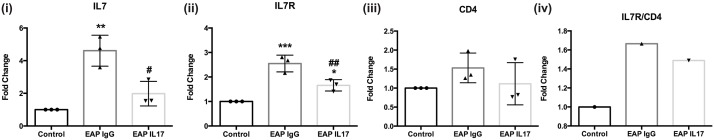
IL17 blockade reduces IL7 expression upon EAP induction. QRTPCR was performed for (i) IL7 and its cognate receptor (ii) IL7R/CD127 and (iii) CD4 on prostate tissues from control, IgG, and anti-IL17 treated EAP mice. (iv) IL7R/CD127 expression normalized against CD4 to examine levels of expression specific to T-cell compartment. Mean +/- SEM are shown, statistics were performed in Prism using one-way ANOVA with Tukey’s multiple comparison tests, (Control vs EAP IgG) * = p<0.05, ** = p<0.01, *** = p<0.001, (EAP IgG vs EAP IL17) # = p<0.05, ## = p<0.01. Experiments performed in technical duplicate and biological triplicate with N = 4/5 mice per group, displayed as average fold change over control value using biological triplicates.

Given the increased expression of IL7 upon EAP induction and the necessity of IL17 for development of pain we next examined the effect of IL17 disruption on IL7 and it’s cognate receptor IL7Ra/CD127. In order to investigate this we performed QRTPCR on cDNA generated from dissociated prostate tissue from mice following, treatment with IgG or anti-IL17 antibody and/or induction of EAP. As expected EAP in tandem with treatment with the control IgG antibody resulted in statistically significant increases in IL7 in prostate tissues compared to untreated mice, Fig 6(i), and treatment with anti-IL17 returned IL7 levels to those observed in control animals. Levels of IL7Ra/CD127 were then assessed in total prostate samples by QRTPCR, Fig 6(ii), and showed similar increases. However, comparing expression levels between untreated controls and anti-IL17 treated mice following EAP revealed that although IL17 blockade reduced IL7Ra/CD127 expression significantly, levels of receptor expression in the prostate remained statistically higher than those observed in control mice. In order to delineate the cellular source of these increases and to determine if IL7Ra/CD127 was induced on CD4+ve T-cells specifically, indicative of differentiation to activated T-cells [24], we performed QRTPCR for CD4 in these tissues, (no differences) Fig 6(iii), and normalized levels of IL7Ra/CD127 against these. Normalization of expression level in such a way revealed that EAP induced T-cell specific expression of IL7Ra/CD127 that could not be reversed by treatment with anti-IL17 alone. It must be noted that these expression data are at the mRNA level and further analyses into the protein level of the IL7Ra/CD127 receptor are needed to fully delineate this model.

## Discussion

This study focused on delineating the immunological mechanism behind chronic prostatitis using a combination of both human patient sample analyses and a murine experimental model. This approach resulted in a more robust understanding of the possible immunological contribution to pain symptoms of the syndrome than had previously been outlined. The role of IL17 in autoimmune disorders is becoming increasingly apparent, as is the plastic nature of T-cell subset differentiation [3,10,11,13,15,34–36]. Furthermore IL17 has been shown to have a role in development of neuropathic and spinal injury induced pain. Direct treatment with recombinant IL17 in certain animal models has shown to be responsible for development of pain and blockade of IL17 using specific antibodies has been shown to be effective in treatment of pain [16,17,37–39]. These processes are heavily dependent on the tissue microenvironment, the presence or absence of certain cytokines, and additional cellular interactions. This report represents the first time that increased levels of CD4+IL17+ cells in the prostate and iliac lymph node tissues of EAP mice has been observed. We posit that this demonstrates the chronic inflammatory nature of EAP and places prostatitis into the growing list of autoimmune disorders, which are mediated to some degree by IL17-regulated inflammation. IL17 has also been associated with the later phases of development of neuropathic pain. In their study, Noma et al examined the expression of IL17 at the level of nerve fibers associated with specific pain models and demonstrated marked increases in expression level up to eight days post peripheral nerve injury [40]. Here however we examined the level IL17 driving pain and autoimmunity in prostate tissues specifically and have not addressed IL17 level associated with neural tissues.

As mentioned, we have previously shown that the CP-1 model of CPPS, results in increases in both IFNγ and IL17 producing CD4+ve T-cells, but that in the absence of IFNγ induction of pain upon bacterial instillation was still observed [3]. Our profiling of the role of IFNγ producing Th1 cells in mediating pain in EAP revealed no observable increase for these cells following 28 days of EAP induction. The data from our anti-IL17 blockade experiments, where no development of pelvic pain occurred in the presence of IFNγ demonstrate that Th1 cells do not play a role in development of pain in the EAP model of CPPS either. The role of FoxP3+ve T-regs in autoimmunity and their intra-plasticity with Th17 cells is an area of active interest in a variety or autoimmune disorders [41,42]. Our data demonstrating increased CD25+FoxP3+ CD4 T-cells in the prostates of anti-IL17 treated mice suggest that this T-cell axis has a role in development of EAP offers the potential for therapeutic intervention in the future. T-regs have also shown to be effective at ameliorating pain and it is thought that skewing towards these anti-inflammatory cells provides novel pathways to treat chronic pain disorders [43].

As mentioned in the introduction, CPPS affects a large diverse group of patients, very few of whom have a documented timeline from when they initially experienced pain to time of diagnoses. Taken together with additional confounding factors such as wide age range, subjective reporting of symptoms, co-morbidity of other syndromes etc., this results in significant difficulty establishing a clean disease population. To overcome these issues we focused on a small population of IIIb patients, who had no history of urinary tract infection or antibiotic use within the last year. These patients also afforded the opportunity to examine the immune context of CPPS patients who are classified as “non-inflammatory”, our contention being that underlying immune dysfunction is a critical mediator of CPPS whether this results in more than 10 leukocytes in EPS samples or not. Interestingly although the IIIa and IIIb disease states are classified as different based on this metric, treatment regimens are not tailored according to these classifiers [44,45]. This argues for the need for a better confluence of patient subjective NIH-CPSI data and objective markers to determine more effective treatments for these patients.

That only one cytokine, IL7, was statistically increased in patients compared to controls and that this cytokine is correlated with every subjective metric used is, we believe, strongly indicative of its role in driving pathology. These data also indicate that perhaps numbers of leukocytic infiltrate in an EPS sample is not representative of the immune environment of prostatitis and may not be the most robust diagnostic tool. IL7 itself is a pro-inflammatory cytokine expressed by a wide variety of cells and tissues, it has been shown to have fundamental role in lymphocyte development [23], has been associated with T1D [11,27,28] and multiple sclerosis [25,26,46–48]. IL7Ra/CD127 has also been implicated in the pathogenesis of multiple autoimmune disorders and it’s blockade using specific antibodies has been shown to mediate the PD-1/PD-L1 self tolerance pathway [49,50] and to decrease incidence of spontaneous T1D in NOD mice. More recently IL7 has been shown to increase the numbers of auto-reactive CD8+ve T-cells while simultaneously attenuating responses that control auto-immunity[29]. The positive correlations observed here are in line with our current hypothesis of CPPS as an autoimmune disorder driven by loss of tolerance to self-antigen of the prostate. The exact mechanism of this loss of tolerance remains an area of current investigation but may involve polymorphisms in either IL7 or IL7Ra/CD127 or perhaps the linked IL2 signaling pathway [23]. GROα, a neutrophilic chemo-attractant, was the only chemokine found to be significantly increased in the EPS of CPPS patients compared to controls. It was not correlated with symptom severity but further implicates IL17 in the pathogenesis of CPPS as it has been shown to be transcriptionally upregulated by the activity of IL17, [51,52]. While we have not demonstrated differences in this cytokine in patients we hypothesize that this may be due to the significant “time to diagnosis” between patients. These variances would dilute out the IL17 expression level changes we would expect to see early in disease initiation and pain development, as it is here that we believe IL17 exerts it’s major effects. Furthermore another chemokine controlled by IL17, MCP-1, while not identified here has previously been shown to be increased in the EPS of CPPS patients [20]. These data further implicate IL17 in the pathogenesis of not only EAP but also human CPPS.

Although IL1RA was negatively correlated with pain scores this was at best a modest correlation and no statistical difference was observed in total levels between patient and control EPS samples. IL1RA has also been shown to play a role in amelioration of pain responses in multiple rodent models of chronic pain disorders, [18,53]. This may however, implicate the IL1 pathway in severity of symptoms rather than in disease onset/mechanism. We also found that as reported pain scores increased the urinary symptom score also increased, and that increased IL7 levels correlated with both symptom metrics. This is interesting given that there is a growing belief that these two symptom scores may be derived from differing mechanisms. The data presented here appear to demonstrate that, in this small IIIb population at least, this is not the case and that increased inflammatory cytokines in EPS of patients may drive both pain responses and urinary dysfunction [45,54–58].

The derivation of Th17 producing T-cells in the context of high IL7 has been shown in additional experimental systems [29,50,59] where IL7, has been shown to control IL17 producing cell maintenance and differentiation [24,60–62]. In studies involving the CD39/CD73/ATP cascade IL7 has been shown to be important for triggering the switch from resting and activated memory suppressive T-cells to Th17 producing pro-inflammatory T-cells. So although we did not identify IL17 itself in the cytokine array there remains rationale for a role for IL17 in human pelvic pain. Data from the anti-IL17 experiments also suggest that T-reg have a role to play in restraining the development of EAP. In this report we demonstrate that blockade of IL17 increases the number of CD4+ve CD25+ve FoxP3+ve T-regs in the prostate of mice following EAP. This, we believe, is indicative of the delicate balance between T-reg and Th17 cells that is central to tolerance to prostatic antigen and/or development of autoimmune prostatitis. Given the development of spontaneous prostatitis in NOD mice, that have defective T-reg responses, and the demonstrated role of IL7 in switching from FoxP3+ve T-regs to IL17 producing cells [27,29], this hypothesis is under active investigation.

Our current working model of prostatitis development posits that damage to the prostate (cytotoxic T-cell (CTL) responses due to sub-cutaneous antigen treatment, or infection with a bacteria (CP-1 model)) induces a Th17 pro-inflammatory response. This initially, is necessary for induction of EAP mediated tactile allodynia but is not sufficient for maintenance of pain. The onset of IL17 mediated inflammation and associated pain is then exacerbated by increased expression of IL7, which serves to promote further maturation of pro-inflammatory T-cells. Controversy surrounds the homeostatic production of IL7, with some studies suggesting that IL7 expression is constitutive with others demonstrating that certain cytokine signals can increase or decrease mRNA levels (e.g. TGF-b, TNF-a) [22,23,63,64], we would favor the latter arguments. Supporting evidence suggests that in such an environment expansion of CD4+ve IL17+ve T-cells, and possible contraction of T-reg function and/or number serves to further exacerbate damage caused by auto-reactive T-cells. This serves to maintain tactile allodynia and allows further breakdown of tolerance.

In conclusion, IL17 expressing Th17 cells are essential for development of pain in the murine EAP model of CPPS and induction of inflammation in this model promotes expression of IL7 a novel immunological marker of symptom severity identified in human EPS samples.

## Supporting Information

S1 Fig Gating used for flow cytometry experiments. A. Gating for lymphocytes based on SSC and FFC in prostate tissues followed by CD4+ve cells followed by either IL17 expression or CD25+veFoxP3+ve cells. Representative staining for control and EAP mouse shown. B. Lymphocytes based on SSC and FFC in prostate tissues then CD4+ve cells and finally IFNγ expression.
(TIFF)

S2 Fig Blockade of IL17 is not sufficient to reduce EAP induced pain. (i) Percentage response frequency increases following EAP development for 10 days followed by treatment with anti-IL17 antibody or IgG control antibody.
(TIFF)

S3 Fig Flow cytometric analysis of T-cell subsets in splenic tissue from control (untreated) and EAP mice. No significant differences were observed between groups for any T-cell marker analyzed. Gating structure was identical to that used in analyses of prostate and iliac lymph node tissue.
(TIFF)

S4 Fig Flow cytometric analysis of T-cell subsets in splenic tissue from control (untreated), IgG treated and anti-IL17A treated EAP mice. No significant differences were observed between groups for any T-cell marker analyzed (CD25, FoxP3, CD4, IFNg and IL17). Gating structure was identical to that used in analyses of prostate and iliac lymph node tissue.
(TIFF)

S1 Table Patient demographics for samples used in EPS multiplex cytokine array. (TIFF)
