# Supplementary figures and images for: IL17 Mediates Pelvic Pain in Experimental Autoimmune Prostatitis (EAP)
Source: PLoS One. 2015 May 1;10(5):e0125623. doi: 10.1371/journal.pone.0125623 (PMC4416802; doi:10.1371/journal.pone.0125623)

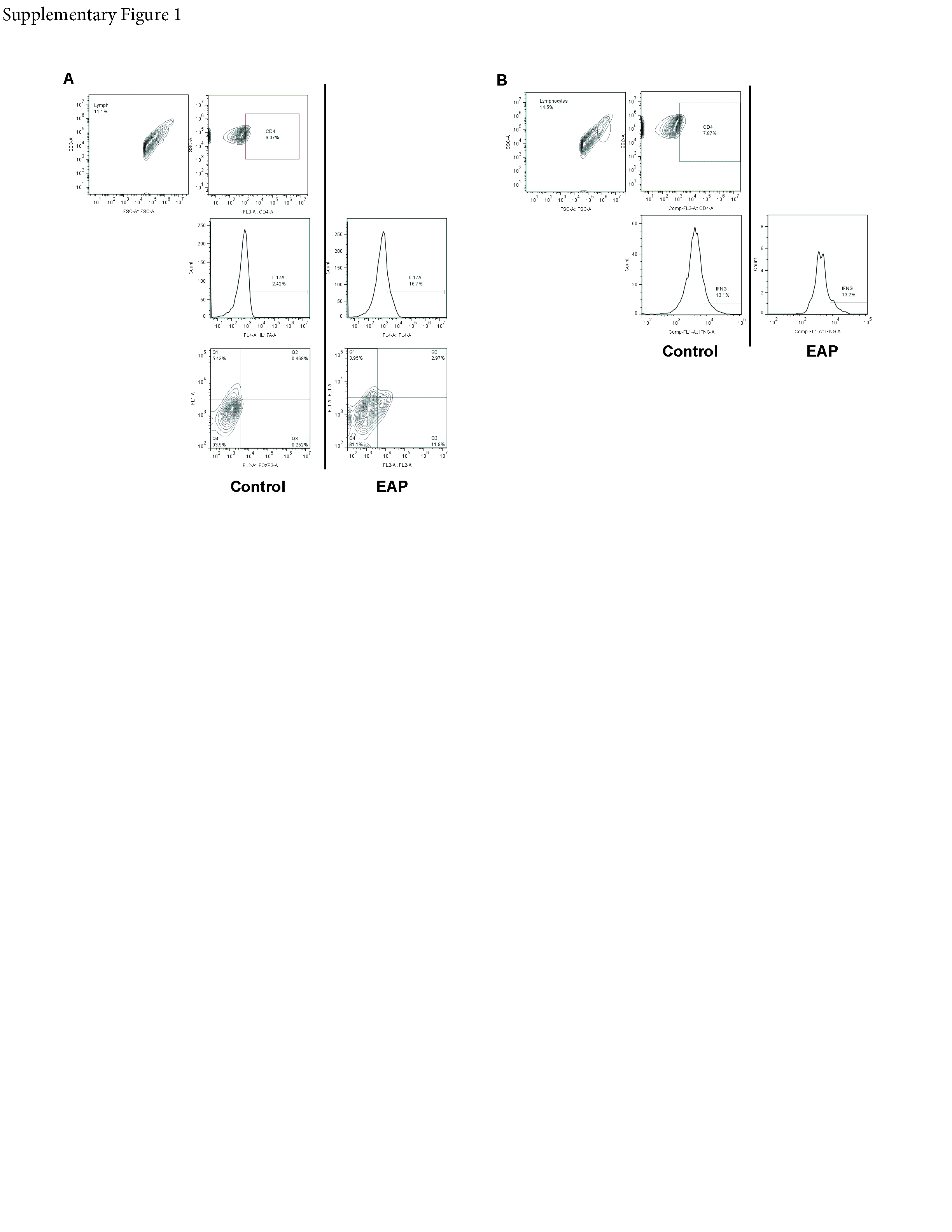

Supplement: S1 Fig — A. Gating for lymphocytes based on SSC and FFC in prostate tissues followed by CD4+ve cells followed by either IL17 expression or CD25+veFoxP3+ve cells. Representative staining for control and EAP mouse shown. B. Lymphocytes based on SSC and FFC in prostate tissues then CD4+ve cells and finally IFNγ expression. (TIFF) [file pone.0125623.s001.tiff]

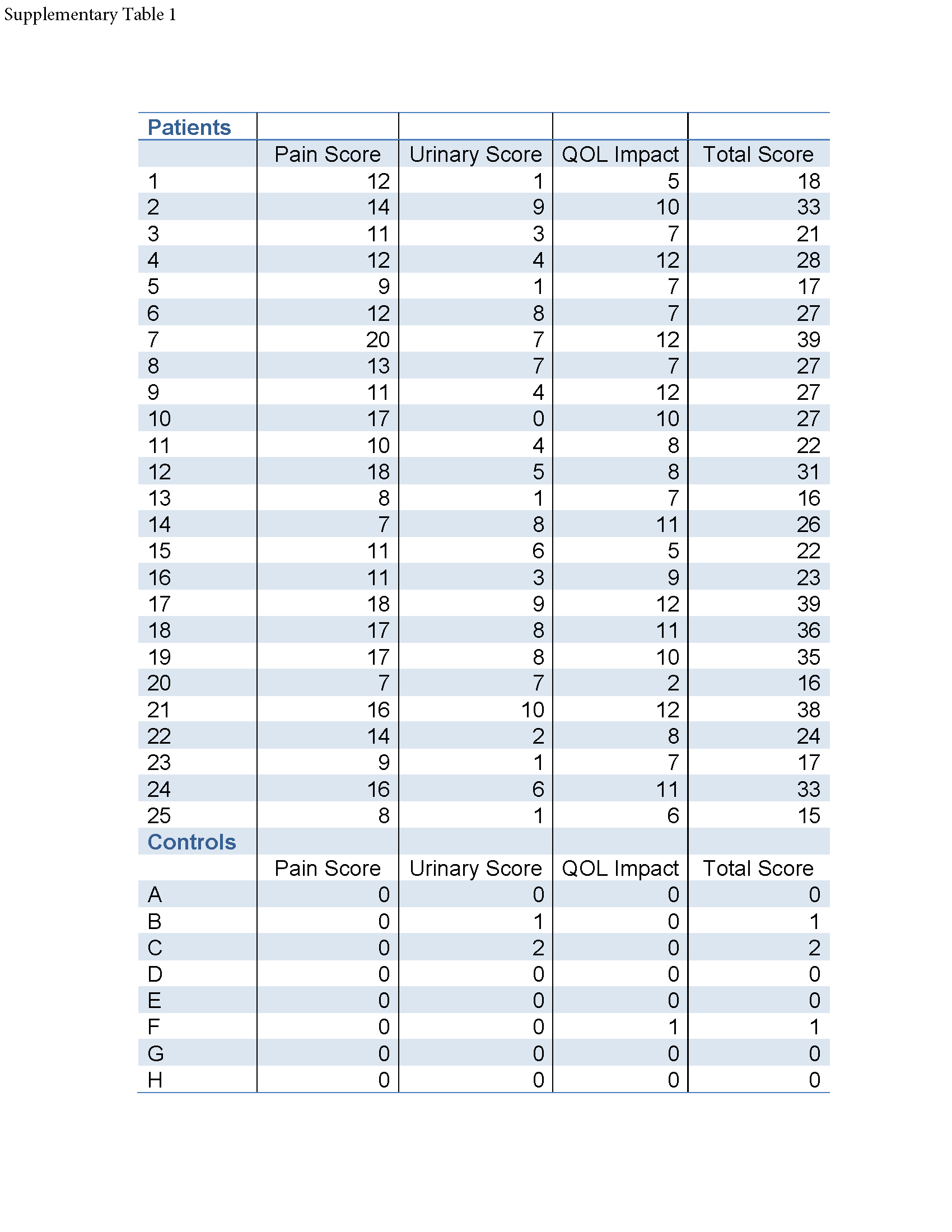

Supplement: S1 Table — (TIFF) [file pone.0125623.s005.tiff]
